# Islet autoimmunity in human type 1 diabetes: initiation and progression from the perspective of the beta cell

**DOI:** 10.1007/s00125-023-05970-z

**Published:** 2023-07-25

**Authors:** Peter J. Thompson, Jasmine Pipella, Guy A. Rutter, Herbert Y. Gaisano, Pere Santamaria

**Affiliations:** 1 Children’s Hospital Research Institute of Manitoba, Winnipeg, MB Canada; 2 Department of Physiology & Pathophysiology, University of Manitoba, Winnipeg, MB Canada; 3 CRCHUM and Department of Medicine, Université de Montréal, Montréal, QC Canada; 4 Department of Diabetes, Endocrinology and Medicine, Faculty of Medicine, Imperial College, London, UK; 5 LKC School of Medicine, Nanyang Technological College, Singapore, Republic of Singapore; 6 Departments of Medicine and Physiology, University of Toronto, Toronto, ON Canada; 7 Cumming School of Medicine, University of Calgary, Calgary, AB Canada; 8 Institut D’Investigacions Biomèdiques August Pi i Sunyer (IDIBAPS), Barcelona, Spain

**Keywords:** Alpha cells, Beta cells, Islet autoimmunity, Review, Type 1 diabetes

## Abstract

Type 1 diabetes results from the poorly understood process of islet autoimmunity, which ultimately leads to the loss of functional pancreatic beta cells. Mounting evidence supports the notion that the activation and evolution of islet autoimmunity in genetically susceptible people is contingent upon early life exposures affecting the islets, especially beta cells. Here, we review some of the recent advances and studies that highlight the roles of these changes as well as antigen presentation and stress response pathways in beta cells in the onset and propagation of the autoimmune process in type 1 diabetes. Future progress in this area holds promise for advancing islet- and beta cell-directed therapies that could be implemented in the early stages of the disease and could be combined with immunotherapies.

**Graphical Abstract:**

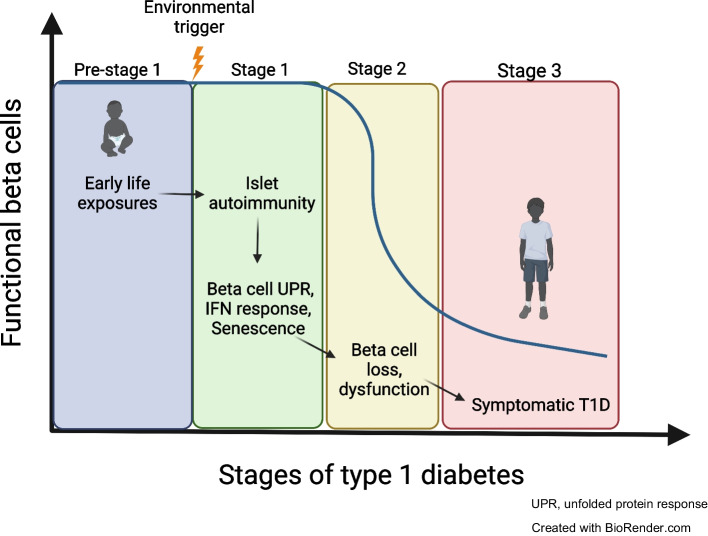

**Supplementary Information:**

The online version contains a slideset of the figures for download, which is available to authorised users at 10.1007/s00125-023-05970-z.

## Introduction

Type 1 diabetes results from organ-specific autoimmunity, which eliminates most of the insulin-producing pancreatic beta cells. While the role of islet autoimmunity remains unquestioned, the role of the islets as active participants has only recently become accepted [1]. It was once believed that virtually all beta cells are lost in type 1 diabetes. However, it now is clear that there is a range of residual beta cells that are dysfunctional in people with newly diagnosed type 1 diabetes [2]. In addition, alpha cell dysfunction is recognised as a prominent feature of the disease [3].

Undoubtedly, sustained efforts by the global type 1 diabetes research community to understand the basis of T cell-driven autoimmunity in type 1 diabetes have paid dividends. The first US Food and Drug Administration-approved immunotherapy for type 1 diabetes, the anti-CD3 antibody teplizumab, delays symptomatic type 1 diabetes onset by 2–3 years [4], opening the door for the future clinical application of antigen-specific approaches with potentially superior immunoregulatory properties [5]. Notwithstanding this achievement, we are still far from a nuanced understanding of how islet autoimmunity originates and propagates during the development of type 1 diabetes and why it is so heterogeneous among individuals. In NOD mice, the following observations have been made: development of type 1 diabetes requires CD4^+^ and CD8^+^ T cells; autoreactive T cells differentiate into killers by engaging beta cell antigens on local antigen-presenting cells; initiating CD4^+^ T cells are insulin-reactive; and CD8^+^ T cells play a major role as beta cell killers [6, 7]. Immunohistopathological studies of pancreases from individuals with type 1 diabetes are generally compatible with these observations made in mice (see references below) but have exposed additional phenomena peculiar to the natural history of the disease in humans. In particular, the dialogue between T cells and beta cells is far more complex in humans and recent evidence supports potential type 1 diabetes ‘endotypes’ based on age at onset [8, 9]. Here, we draw upon studies that have shed light on the initiation and progression of islet autoimmunity from the point of view of the beta cell. We advance the hypothesis that antigen presentation by beta cells, their stress responses and functional heterogeneity are critical factors that will provide clues towards solving the enigma posed by islet autoimmunity in type 1 diabetes.

## How is islet autoimmunity initiated? The chicken and the egg

### Autoantibody positivity

Central to solving the puzzle as to how type 1 diabetes occurs will be to identify the elusive cause(s) of initial islet autoimmunity. The concept that islet autoimmunity results from genetic susceptibility combined with an environmental trigger, first theorised by Eisenbarth [10], is now generally accepted [11]. Symptomatic, or ‘stage 3’ type 1 diabetes, generally occurs following two clinically tractable stages consisting of seroconversion and the presence of two or more autoantibodies, followed by development of dysglycaemia [11]. While valuable clinically, the model has some limitations, some of which are just emerging. For example, the single-autoantibody-positive stage generally has a significantly lower of risk of progression [12] and as such is not considered as the earliest stage of type 1 diabetes. Nevertheless, recent work has revealed defects in glucagon secretion in islets isolated from donors positive for a single GAD autoantibody (GADA) [13]. Furthermore, all autoantibodies are not equal in terms of risk, and the order of autoantibody appearance influences progression to type 1 diabetes [14]: 22% of single-GADA-positive individuals progress to multiple autoantibodies; and about 6% of those with multiple antibodies progress to stage 3 symptomatic type 1 diabetes [15]. On the other hand, there is heterogeneity between those individuals that develop multiple autoantibodies, with many not progressing to type 1 diabetes [16]. Regardless, in terms of initiation of islet autoimmunity, the model posits an environmental triggering event of some kind.

### Benign autoimmunity

Although insulin is only one of many type 1 diabetes-relevant autoantigens, several lines of evidence suggest that insulin autoreactivity, both in mice and humans, plays a key role in the initiation of type 1 diabetes [17, 18]. Mounting evidence also suggests that diabetogenesis involves CD4^+^ T cell recognition of hybrid peptides generated by fusion of insulin to other autoantigens such as chromogranin A and islet amyloid polypeptide (hybrid insulin peptides [HIPs]) [19]. Although it remains unclear why and how these T cells become activated, an extra-pancreatic priming event triggered by systemic delivery of the beta cell-generated insulin peptides is a likely possibility [20]. In turn, it seems reasonable to propose that priming of the disease-initiating T cell specificities is preceded by quantitatively and/or qualitatively abnormal exposure of these T cells to cognate insulin epitopes.

In agreement with these considerations, a growing body of evidence supports the view that most if not all people harbour autoreactive T cells in the peripheral repertoire, regardless of their autoimmune disease proclivity [21]. While this is inconsequential in most individuals, beta cell insults coupled to genetic predisposition could trigger the activation and recruitment of these T cells to target organs, such as the pancreatic islets in type 1 diabetes. For example, CD8^+^ T cells recognising epitopes from several beta cell autoantigens circulate at similar frequencies in both individuals with type 1 diabetes and non-diabetic individuals but are exclusively recruited to the pancreas in individuals with type 1 diabetes, where they exhibit an antigen-experienced memory phenotype [22]. Coppieters et al [23] demonstrated the presence of various beta cell autoreactive CD8^+^ T cell specificities in pancreases from a subset of individuals up until 8 years post diagnosis, highlighting the chronic and heterogeneous nature of the autoimmune attack underlying the progression of type 1 diabetes. In a similar study, Bender et al [24] used in situ tetramer staining to demonstrate that preproinsulin-specific CD8^+^ T cells are found in healthy donor pancreas tissue in similar proportions to those in autoantibody-positive stage 1–2 donors and donors with recent-onset type 1 diabetes. In healthy donor pancreases, these self-reactive CD8^+^ T cells appear to remain in a seemingly dormant state, dispersed in the exocrine compartment. However, in pancreases from donors with type 1 diabetes, these CD8^+^ T cells are much more frequently observed in contact and proximity with insulin-containing islets and display features of antigen experience [24]. Similarly, a higher frequency of circulating preproinsulin-specific CD8^+^ T cells exhibit an antigen-experienced memory phenotype in recent-onset type 1 diabetes and in stage 2 autoantibody-positive children, as compared with healthy control individuals [25]. Together, these studies suggest that initiation of islet autoimmunity may require beta cell insults capable of eliciting the activation and recruitment of autoreactive T cells to the pancreas. Genetically imprinted inter-individual differences in the avidity of specific autoreactive T cells, differences in absolute autoreactive T cells frequencies, and/or differences in T cell activation thresholds, which are impacted by thymic selection processes, can help determine the likelihood and the extent to which one or more ‘environmental’ triggers’ may lead to chronic autoimmunity and eventually beta cell destruction [26].

### Environmental triggers

Putative environmental triggers for islet autoimmunity in type 1 diabetes remain elusive (reviewed in [27]) but molecular mimicry between beta cell and viral antigens [22, 28] may be a plausible link that explains the selective activation of islet-reactive T cells in certain individuals. Epidemiological studies of large prospective paediatric cohorts have provided insights into early life exposures associated with seroconversion, progression to multiple autoantibodies and finally symptomatic type 1 diabetes [29, 30]. The BABYDIAB and BABYDIET studies established that in genetically susceptible newborns the peak incidence of seroconversion (development of the first islet autoantibody) occurs between 9 months and 2 years of age [29], and children who develop multiple autoantibodies before age 3 years are much more likely to progress to type 1 diabetes in the ensuing 10 years as compared with those who do not [30]. Recent updates from The Environmental Determinants of Diabetes in the Young (TEDDY) study support the view that the development of islet autoimmunity is associated with chronic enteroviral B infection [31]. New findings from the Diabetes Virus Detection (DiViD) study lend further evidence implicating live enteroviruses in new-onset type 1 diabetes [32]. In the first study of its kind to demonstrate live virus in the pancreas of living individuals with type 1 diabetes, the authors found that resected pancreas tail tissue from 6/6 individuals recently diagnosed with type 1 diabetes (within 1 month of diagnosis) harboured enterovirus, whereas only two out of 11 from normoglycaemic/non-diabetic individuals did [32]. Remarkably, other viruses were much less frequently found in the diabetic pancreases [32], arguing against a general predisposition to viral infection in people with type 1 diabetes. While a potential environmental triggering event could plausibly consist of persistent enteroviral infection, it is important to note that previous studies have not found a correlation between enteroviruses and type 1 diabetes (extensively reviewed in [33]) and it is unlikely that the ensuing heterogeneity in the progression of islet autoimmunity could be explained by a single kind of environmental trigger in all cases. Future investigations on individuals at early presymptomatic stages of type 1 diabetes will be required to establish whether chronic enteroviral infections could selectively promote activation of beta cell-specific T cell populations. Additional studies should also consider events even earlier in life, such as in utero sensitising events, and their impact during the window of beta cell development in the fetus. Indeed, recent evidence from the TEDDY study showed a correlation between the number and type of islet autoantibody and various forms of maternal psychosocial stress during pregnancy [34].

### Early life metabolic changes

But is islet autoimmunity merely a matter of immune-activating events or do earlier clinically tractable events occur in the islets/beta cells? A recent study by Warncke et al [35] sheds light on this vital question for the first time through a comprehensive analysis of pre- and postprandial glucose levels in a large cohort of infants prior to seroconversion within the critical window of islet autoimmunity development. This study analysed over 5000 preprandial and over 3000 postprandial blood glucose measurements in a prospective cohort of over 1000 infants and toddlers from 4 months to 3.5 years of age as part of the Primary Oral Insulin Trial (POInT) [36]. Remarkably, the study demonstrated that there are subtle but significant increases in 30 min postprandial blood glucose in infants (from 4 months to 1.5 years of age) that experienced seroconversion to single or multiple autoantibodies as compared with those that did not [35]. At toddler stages (1.5–3.5 years) differences were observed in preprandial blood glucose between the groups. Seroconversion was detected at a median age of 1.8 years but postprandial blood glucose levels did not differ between infants that went on to develop islet autoantibodies vs those that did not, just up until 2 months prior to seroconversion [35]. Strikingly, these data suggest that alterations in glucose metabolism and/or beta cell function precede the development of islet autoimmunity [35] and it is tempting to speculate that early life alterations in beta cell function could play a causal role in islet autoimmunity. There are important limitations to consider in this study, since at present it is unclear whether autoimmune initiation is the same as seroconversion. Nevertheless, this study furnishes a new perspective on the role of early postnatal development in shaping the functioning and responsiveness of islets and their interactions with the immune system in humans. A body of evidence from studies in rodents highlights the importance of diet interactions with the epigenome in neonatal beta cells (reviewed in [37]) and supports the importance of the neonatal period in laying the foundations for the long-term health of beta cells in adults. As very few studies have interrogated the mechanisms of human beta cell development and function in the neonatal period [38–40], further work in this area is urgently needed. Major consortia, such as the US NIH-funded Human Atlas of Neonatal Development & Early Life Pancreas (HANDEL-P) (https://www.pancreatlas.org/datasets/531/overview, accessed 23 June 2023), have the resources to make advances in this field on behalf of the community.

## How does islet autoimmunity progress? A role for antigen presentation by beta cells

### Beta cells and the progression of islet autoimmunity

The initiation of islet autoimmunity is likely to involve both early life developmental changes in beta cells as well as environmental exposures but what factors drive the further progression and development of islet autoimmunity? Islet autoimmunity dynamically evolves and expands during the progression from stage 1 to stage 3 type 1 diabetes, as evidenced by the presence of increasing numbers of autoantibodies and autoreactive T cells specificities. Islet-reactive T cells recognise an increasing number of beta cell antigens and epitopes as the disease progresses, consistent with both antigen and epitope spreading [41]. Thus, dynamic antigen presentation by beta cells may be a driving factor in the progression of islet autoimmunity (reviewed in [42]). It is well established that human islets overexpress HLA Class I molecules during early stages of type 1 diabetes [43]; however, studies now report expression of HLA Class II molecules on beta cells in type 1 diabetes as well [44, 45]. Elegant immunopeptidomics of HLA Class I from human beta cell lines and tetramer staining confirms that beta cells present both conventional and modified antigens to islet-infiltrating CD8^+^ T cells [46, 47]. Likewise, studies of human islet-reactive CD4^+^ T cells have shown that at least some of the beta cell-derived epitopes that are targeted by diabetogenic CD4^+^ T cells arise from alternative splicing of antigen-coding mRNAs or from the post-translational fusion of fragments of insulin and other secretory granule hormones, the so-called HIPs [48, 49]. T cells recognising these antigenic targets would readily escape central tolerance, and hence play a more significant, albeit not exclusive, role in diabetogenesis owing to their higher avidity for cognate peptide major histocompatibility complex (pMHC) (in the absence of thymic censorship). One of the main take-home messages from this body of work is that conventional and typically processed autoantigens only represent the tip of the iceberg in terms of antigen complexity during the progression of islet autoimmunity. Preproinsulin and insulin (or derivatives) are major antigenic targets of the human autoreactive T cell repertoire that infiltrate islets in type 1 diabetes [17, 50]. Post-translational modification, alternative splicing, hybrid-fusion, defective ribosomal products and other unconventional beta cell antigens have all been shown to underlie the activation of at least some autoreactive T cells responses. These unconventional beta cell autoantigens are collectively referred to as ‘neoantigens’ (reviewed extensively elsewhere [28]), since they comprise immunologically novel sequences or structures that are selectively expressed (and exposed to autoreactive T cells) in the islet niche. As antigen presentation by beta cells is greatly altered by various forms of stress (reviewed in [51] and further detailed below), stress pathways operating in beta cells are a major cause of neoantigen formation.

### Beta cell death and islet autoimmunity

Another major unanswered question concerns the relationship between beta cell death and islet autoimmunity progression. As the mode and kinetics of beta cell death during the presymptomatic stages of type 1 diabetes clearly vary between people, how this influences the evolution of antigen presentation and neoantigen formation remains to be determined. Neiman et al [52] measured cell-free beta cell DNA (identified by its beta cell-specific methylation profile at key genes) as a surrogate for beta cell death in a cohort of autoantibody-positive individuals (*n*=32), individuals with very recent-onset type 1 diabetes (<4 months *n*=92), individuals with long-standing type 1 diabetes (>4 months, *n*=38) and normoglycaemic/healthy control donors. The authors surprisingly found no significant differences in circulating beta cell DNA in the autoantibody-positive donors vs donors with type 1 diabetes relative to controls [52]. Despite some limitations with the sensitivity of the assay and the cross-sectional nature of the study, a significant difference in cell-free beta cell DNA was found in donors who had received an islet transplant compared with healthy donors, likely reflecting the extensive beta cell death that frequently occurs post transplant [52]. Thus, whatever conclusions can be drawn from this study concerning the mode or kinetics of beta cell loss during type 1 diabetes (whether it is progressive, relapsing–remitting, etc.), beta cell death during the early and symptomatic stages of type 1 diabetes is unlikely to release large quantities of cell-free DNA or antigens, as occurs during islet transplantation. It therefore remains to be determined how the mode and kinetics of beta cell death affect the evolution of beta cell antigenicity and neoantigen formation during the development of type 1 diabetes.

### Lessons from mouse models

The view that antigen presentation by beta cells is critical for progression of islet autoimmunity has been supported by mechanistic studies manipulating genes in beta cells in the NOD mouse model. Conditional knockout of the unfolded protein response (UPR) mediator *Ire1α* (also known as *Ern1*) in young beta cells of NOD mice leads to a dedifferentiated phenotype, which alters antigen presentation and halts disease progression [53]. Adoptive transfer of cytotoxic T cells from mice with beta cell-specific *Ire1α* deletion failed to induce diabetes in recipient mice [53], revealing a fundamental phenotypic change in autoimmunity precipitated by the alterations in the beta cells. Similarly, mutation of the type 1 diabetes genome-wide association study gene *Rnls*, which controls UPR signalling, led to reduced recognition of NIT-1 insulinoma beta cells by polyclonal autoreactive T cells from diabetic NOD mice, without affecting MHC class I or II expression [54]. Enforced early proliferation of young beta cells in NOD mice by liver insulin receptor knockout (LIRKO) before immune cell infiltration led to profound changes in antigen presentation concomitant with a diminution of autoreactive T cell activity [55]. NOD mice harbouring *Ins1-Cre* or a reduced dose of insulin genes show a lower diabetes penetrance but no major changes in autoantibodies or insulitis [56], and swapping the mouse *Ins1* gene for the human *INS* gene also has a protective effect in NOD mice [57]. Given the role of insulin as a major autoantigen in type 1 diabetes, the findings from these latter studies are also consistent with altered/reduced presentation of insulin antigens, leading to slower autoimmunity progression and lower type 1 diabetes penetrance in NOD mice. While care should be taken in extrapolating the relevance of results in NOD mice to humans, this work highlights the dynamic relationships between antigen presentation by beta cells and autoimmunity and in particular the significance of beta cell maturation stage.

## Beta cell stress responses and islet autoimmunity

**Fig. 1:**
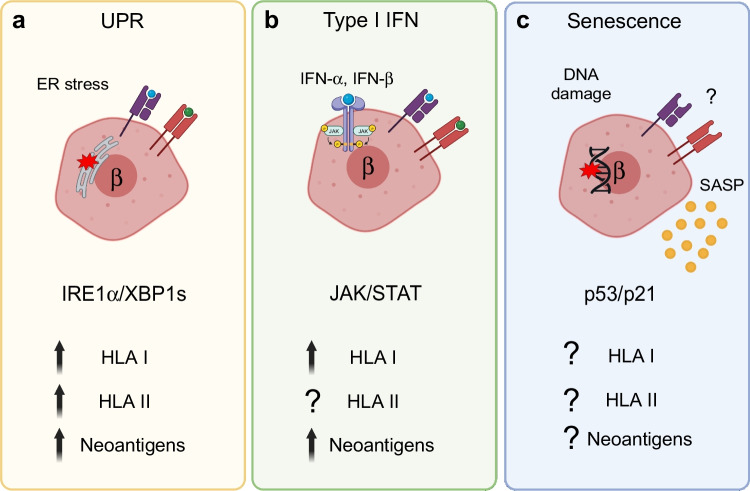
Human beta cell stress responses during the development of type 1 diabetes and effects on antigen presentation. (**a**) ER stress activates the UPR, which either restores homeostasis (adaptive) or initiates apoptosis (terminal). Activation of the UPR may alter antigen presentation via changes in HLA Class I and II expression and formation of neoantigens. (**b**) Type I IFN response is triggered by cytokines IFN-α and IFN-β and leads to activation of JAK/STAT signalling. The IFN response upregulates HLA Class I and II expression and could also lead to the formation of neoantigens via effects on alternative splicing, among other mechanisms. (**c**) Senescence is a stress response characterised by DNA damage, activation of the p53/p21 pathway and an inflammatory secretome (SASP). It is currently unclear how senescence could alter human beta cell antigen presentation but evidence from NOD mice [61] suggests an impact on Class I and II expression, antigen processing machinery and potential for neoantigen formation. β, beta cell. Created with BioRender.com. This figure is available as part of a downloadable slideset

A growing number of studies support the view that beta cell stress responses, including the UPR, type I IFN response and senescence, contribute to the progression of islet autoimmunity and type 1 diabetes (reviewed in [58]). Each of these stress responses occur in human beta cells in type 1 diabetes as shown by immunohistological studies on donor pancreas [59–61]. Furthermore, drug-based interventions that mitigate each of these stress responses appears to be sufficient to halt diabetes progression in NOD mice (reviewed in [62]). Moreover, results from clinical trials targeting the UPR in individuals with new-onset type 1 diabetes [63, 64] provide support for the translational potential for targeting beta cell stress in humans. Below, we detail each of these stress pathways and what has been learned about their relationships with islet autoimmunity (Fig. 1).

### Beta cell UPR

Beta cells are prone to endoplasmic reticulum (ER) stress as a general consequence of their professional secretory functions [65]. ER stress activates the UPR, a three-branch signalling cascade that functions to re-establish cellular homeostasis and alleviate ER stress. The three main UPR sensors are inositol requiring enzyme 1 (IRE1), protein kinase R-like endoplasmic reticulum kinase (PERK) and activating transcription factor 6 (ATF6). According to the canonical model of the UPR system in beta cells, if the adaptive UPR fails to reduce chronic ER stress, a terminal UPR occurs, which triggers apoptosis [66]. Activation of UPR either via chemical stressors or inflammatory cytokines upregulates HLA Class I and II expression, alters antigen presentation by beta cells and elicits neoantigen formation in both mouse and human islets/beta cells [45, 46, 67]. In addition, UPR activation with thapsigargin increases human induced pluripotent stem cell beta cell HLA Class I antigen presentation to type 1 diabetes donor T cells in vitro in an autologous co-culture system [68]. Most of our knowledge of the UPR in beta cells during type 1 diabetes and its relationship with antigen presentation comes from work on the IRE1α/spliced x-box binding protein 1 (XBP1s) signalling arm [66] (Fig. 1a), where changes in this pathway have been linked with altered antigen presentation [53, 67]. However, it is not entirely clear whether other UPR mediator(s) also play a role and how sustained the effects on antigen presentation are; the balance between adaptive and terminal UPR may play a significant role.

### Beta cell type I IFN response

The type I IFN response is a broadly acting cellular antiviral defence. IFN-α and IFN-β activate janus kinase/signal transducers and activators of transcription (JAK/STAT) pathway signalling leading to a variety of different outcomes, including IFN-stimulated gene expression, ER stress, HLA Class I overexpression and eventual beta cell apoptosis in human donor islets ex vivo and in the human beta cell line EndoC-βH1 [69]. This stress response also shows a number of genetic associations with type 1 diabetes [70]. Although upregulation of HLA Class I is observed throughout the islet in type 1 diabetes [43], other markers of the IFN response occur preferentially in islets with remaining beta cells and insulitis in autoantibody-positive pancreas donors and those with type 1 diabetes [71]. While treatment with inflammatory cytokines IFN-γ, IL-1β and TNF-α also triggers HLA Class II upregulation in human islet beta cells [45], whether a similar response occurs following IFN-α or IFN-β stimulation has not been addressed (Fig. 1b). Interestingly, the RNA editing enzyme adenosine deaminase RNA specific 1 (ADAR1) is upregulated in response to IFN-α but antagonises the IFN pathway by A-to-I editing and alternative splicing of human beta cell mRNAs, thereby altering the transcriptome and potentially leading to neoantigens [72]. These results highlight the diverse mechanisms by which type I IFN signalling can impact antigen presentation by beta cells and thus could influence progression of islet autoimmunity.

### Beta cell senescence

In contrast to both UPR and type I IFN responses both of which can trigger apoptosis, beta cell senescence is a stable anti-apoptotic stress response that involves growth arrest and a proinflammatory secretome termed the senescence-associated secretory phenotype (SASP) [62]. Single-cell RNA-seq suggested that senescent beta cells in NOD mice upregulate a variety of antigen processing and presentation genes, including MHC I and II [61], but whether these changes are reflected at the protein level was not determined. Similarly, it has not been determined whether senescence in human islet beta cells elicits changes in HLA Class I or II expression or antigen presentation (Fig. 1c). Due to SASP and apoptosis resistance, senescent beta cells could persist to continue a long-lasting dialogue with the immune system. Though key details remain to be explored, further investigations into how beta cell senescence alters antigen presentation are clearly warranted.

## Do individual beta cells differ in their susceptibility to islet autoimmunity?

### Beta cell heterogeneity and islet autoimmunity

The notion of beta cell functional heterogeneity is increasingly accepted as a natural and biologically relevant feature of islet architecture and health [73, 74]. Salomon and Meda first demonstrated the existence of rodent beta cell populations with differing secretory activities [75] and a few years later, Pipeleers and others [76, 77] showed beta cell subpopulations that varied in their metabolic responses to glucose. Transcriptomic and other data (reviewed in [78]) have now reinforced this view.

An important feature of heterogeneity in islet function is the connectivity between individual beta cells, resulting at least in part from the existence of gap junctional coupling. Supporting the importance of this process, inactivation of the gap junction protein connexin 36 (encoded by *Gjd2*) in beta cells results in defective insulin secretion and glucose tolerance in mice [79, 80]. Additionally, highly connected ‘hubs’, representing ~5% of beta cells, that host a disproportionate number of connections to other cells [81] are involved in the transmission of Ca^2+^ signals across the mouse islet. These phenomena are predicted by modelling to play an important role in islet cell physiology [82, 83] and appear also to be important in human islets [82]. Of note, different islets display discrete wave types and behaviours, with ‘hub’ behaviour most prominent in islets displaying more rapid Ca^2+^ oscillations [84]. At least for the functionally defined leader and hub beta cells, respectively, the existence of discrete transcriptomes [84] and of protein markers including glucokinase [81] argues that these reflect stable subsets of cells rather than stochastic, functional ‘states’. The relevance of this inter-islet heterogeneity to both normal insulin secretion and type 1 diabetes pathogenesis, is difficult to study given the challenges of interrogating the function of multiple islets while preserving their spatial interrelationships simultaneously within the intact pancreas but may be achievable in pancreatic slice preparations, at least over relatively short ranges (a few mm). Genes involved in modulating beta cell function may also contribute to the hub/leader–follower dynamic. Of particular interest in this context are those genes implicated, through genetics, in the pathogenesis of both type 1 diabetes and type 2 diabetes [85], such as human *GLIS2* and *PTPN2*, as well as genes shown in our mouse studies to be enriched (*Gck*) or depleted (*Ins*) in hub cells, or to be enriched (e.g. *ADCY6*) in leader cells [84]. Single-cell RNA-seq data from individuals with type 1 diabetes and non-diabetic control individuals from the Human Pancreas Analysis Program (HPAP) reveal changes in transcript levels in both beta and alpha cells, with immune-related genes strongly represented in both cell types [86].

**Fig. 2:**
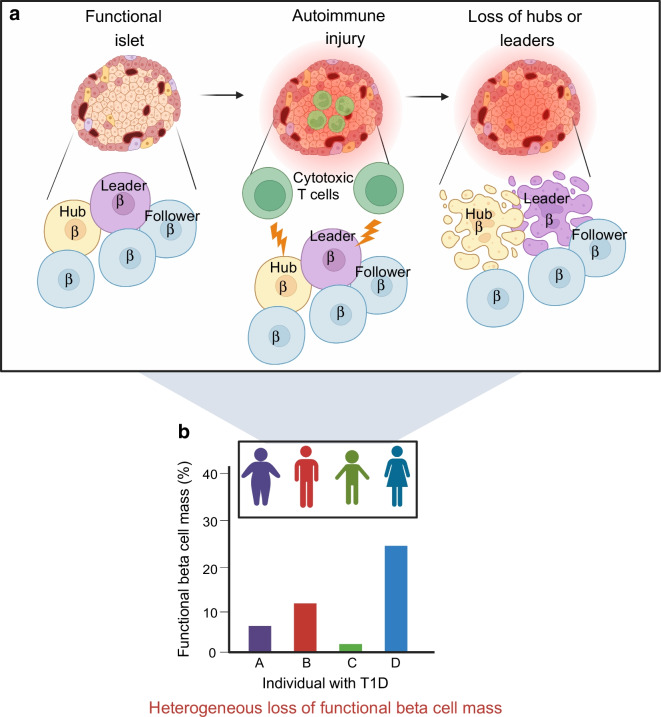
Heterogeneity in the autoimmune response in type 1 diabetes and its potential relationship with inter-individual heterogeneity in residual beta cell mass. (**a**) Islet function is heterogeneous and insulin secretion from the islet is controlled by a small subpopulation of ‘hub’ and ‘leader’ beta cells that coordinate the action of ‘follower’ cells. (**b**) Preferential autoimmune destruction of specialised hub or leader beta cells could help to explain the observation of inter-individual variation in residual functional beta cell mass in individuals with type 1 diabetes. β, beta cell; T1D, type 1 diabetes. Created with BioRender.com. This figure is available as part of a downloadable slideset

Whether leader or hub (or other) specialised beta cells, or islets enriched in these ‘regulatory’ cell types, are preferentially targeted in type 1 diabetes is not known (Fig. 2). Type 1 diabetes-related stressors, such as cytokine treatment, lower the apparent number of hubs in mouse islets [87] but whether this reflects loss or dysfunction of these cells or weakening of the connections between these and subservient followers is unclear. Likewise, it is unknown whether these specialised beta cell subtypes have any relationship to beta cells that undergo stress responses, such as UPR or senescence, during type 1 diabetes. In any case, preferential loss of leaders or hubs may conceivably lead to islet dysfunction (where a significant number of ‘dormant’ beta cells remain [88]), in addition to beta cell death, in type 1 diabetes [89]. Future studies to test this hypothesis will involve quantifying leader and hub cell numbers during the natural history of type 1 diabetes. This may involve live cell analysis in vitro (e.g. using pancreatic slice preparations) or in vivo after engraftment into the anterior eye chamber of immune-compromised mice. An alternative and attractive possibility given their availability (e.g. through the Network for Pancreas Organ Donors with Diabetes, nPOD), is the analysis of fixed samples from individuals with type 1 diabetes. Examination of the mechanisms involved in any selective cell loss, such as preferential expression of conventional or neoantigens, might then be facilitated by prospective studies in vivo [90, 91].

## Alpha cells and the initiation and progression of islet autoimmunity

Alpha cells in the human islet are much more abundant than in rodents (30–50% of the islet, a value which approaches or may even exceed that of beta cells in some islets) [92]. During the natural history of type 1 diabetes, alpha cells develop dysfunction including failure of high-glucose-mediated suppression and poor response to low glucose (hypoglycaemic blindness) induced by iatrogenic insulin administration [93]. It was initially thought that the alpha cell becomes dysregulated only in later stages of type 1 diabetes when loss of beta cell mass (leading to deficient insulin and unrestrained somatostatin secretion) perturbs paracrine modulation of glucagon release (reviewed in [94]) [95]. However, reports have shown early loss of the glucagon response to hypoglycaemia in type 1 diabetes [96]. In fact, the beta cells of islets isolated from single GADA-positive normoglycaemic donors [13], and those surviving in type 1 diabetes, have remarkably preserved secretory responses [97]. In contrast, alpha cells from islets isolated from normoglycaemic GADA-positive donors and donors with type 1 diabetes show impaired suppression of glucagon secretion by glucose [13, 97]. This would indicate that both intrinsic and extrinsic defects affect the alpha cell in type 1 diabetes.

### Alpha cells and the progression of islet autoimmunity

Whereas T cell-mediated destruction of beta cells is well-studied, the collateral damage of this process on alpha cells in the islet microenvironment is poorly understood. A subset of islet-infiltrating T cells recognise peptides derived from proglucagon, and serum autoantibodies to glucagon have been reported in the NOD mouse model [98], although current evidence has shown this does not occur in humans [17]. Regardless of the possible mechanisms involved, it is becoming clear that approaches to restore alpha cell function have a beneficial effect on beta cell health and survival in type 1 diabetes. Recent studies targeting glutamate [99] and glucagon [100] receptors have demonstrated remarkable efficacy on human type 1 diabetes pancreas slices and type 1 diabetes mouse models in normalising glucagon secretion and improving beta cell mass, respectively. Future studies are required to delineate how aberrant alpha cell function early in type 1 diabetes might influence the progression of the autoimmune attack on beta cells and whether targeting of alpha cell dysfunction could be a viable therapeutic option to ameliorate the progression of islet autoimmunity and/or to preserve beta cell mass and function in humans.

## Conclusion

In conclusion, there are many remaining questions surrounding the origins of islet autoimmunity and how it develops during type 1 diabetes. We are only beginning to appreciate the roles of islets and of beta cells themselves in autoimmunity. Early beta cell development as well as the metabolic, phenotypic, transcriptomic and epigenetic reprogramming that beta and alpha cells undergo during diabetogenesis remain poorly understood in humans. Studies within the critical window when seroconversion frequently occurs in infants with high genetic risk will provide key mechanistic insights. We still have much to learn about how beta cell antigen presentation, stress pathways, functional heterogeneity and alpha cells shape the autoimmune response in type 1 diabetes. Sustained progress in this area will reveal the mechanisms underlying heterogeneity in islet autoimmunity and guide our efforts to move clinical interventions in type 1 diabetes towards precision medicine.

### Supplementary Information

Below is the link to the electronic supplementary material.

Supplementary file1 (PPTX 506 KB)

## Abbreviations

ER: Endoplasmic reticulum
GADA: GAD autoantibody
HIP: Hybrid insulin peptide
SASP: Senescence-associated secretory phenotype
TEDDY: The Environmental Determinants of Diabetes in the Young
UPR: Unfolded protein response
