# Supplementary material for: Islet autoimmunity in human type 1 diabetes: initiation and progression from the perspective of the beta cell
Source: Diabetologia. 2023 Jul 25;66(11):1971–82. doi: 10.1007/s00125-023-05970-z (PMC10542715; doi:10.1007/s00125-023-05970-z)
Supplement: Supplementary file 1 — Supplementary file1 (PPTX 506 KB) [file 125_2023_5970_MOESM1_ESM.pptx]

## Slide 1
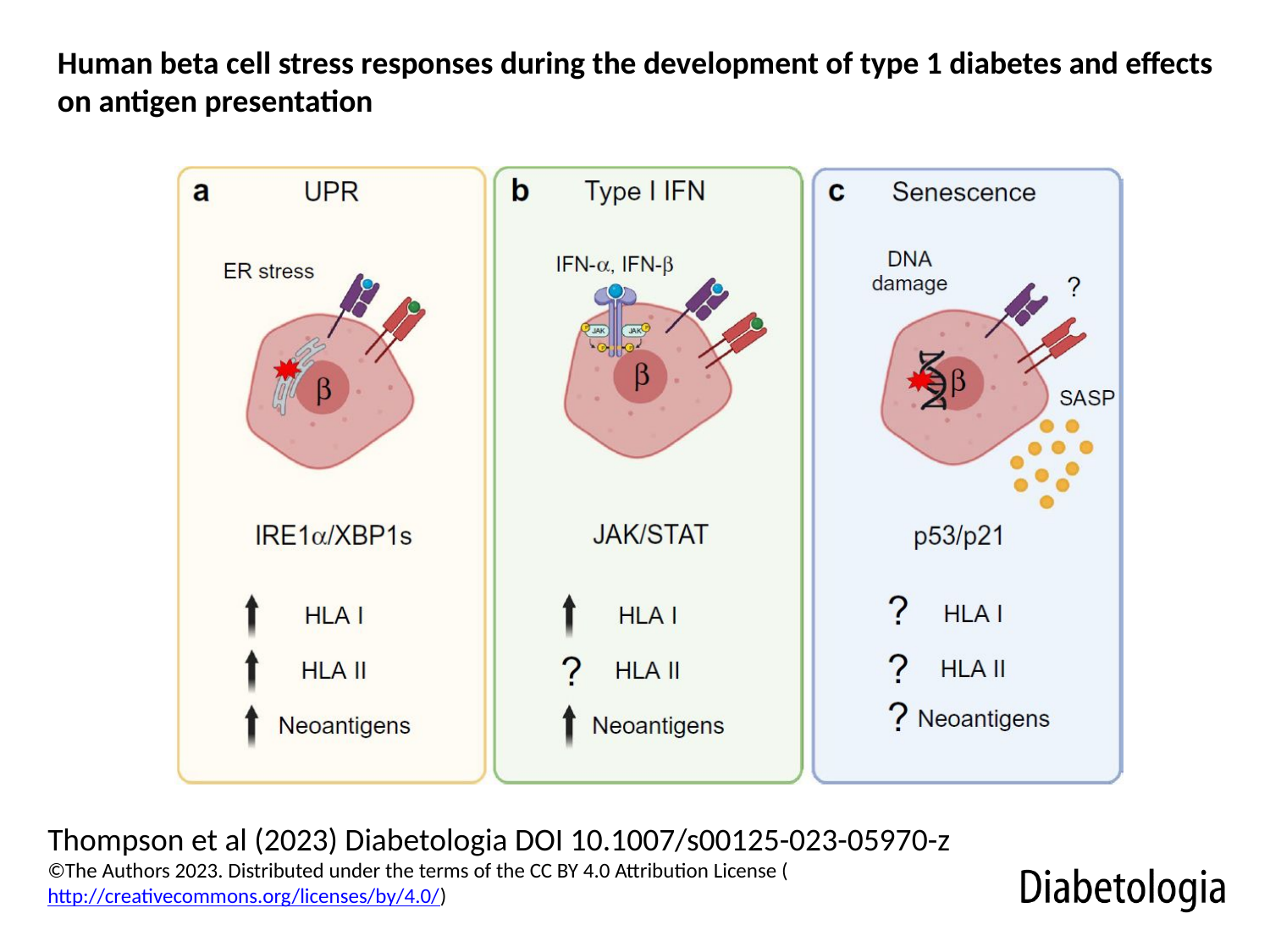

Human beta cell stress responses during the development of type 1 diabetes and effects on antigen presentation
Thompson et al (2023) Diabetologia DOI 10.1007/s00125-023-05970-z
©The Authors 2023. Distributed under the terms of the CC BY 4.0 Attribution License (http://creativecommons.org/licenses/by/4.0/)

## Slide 2
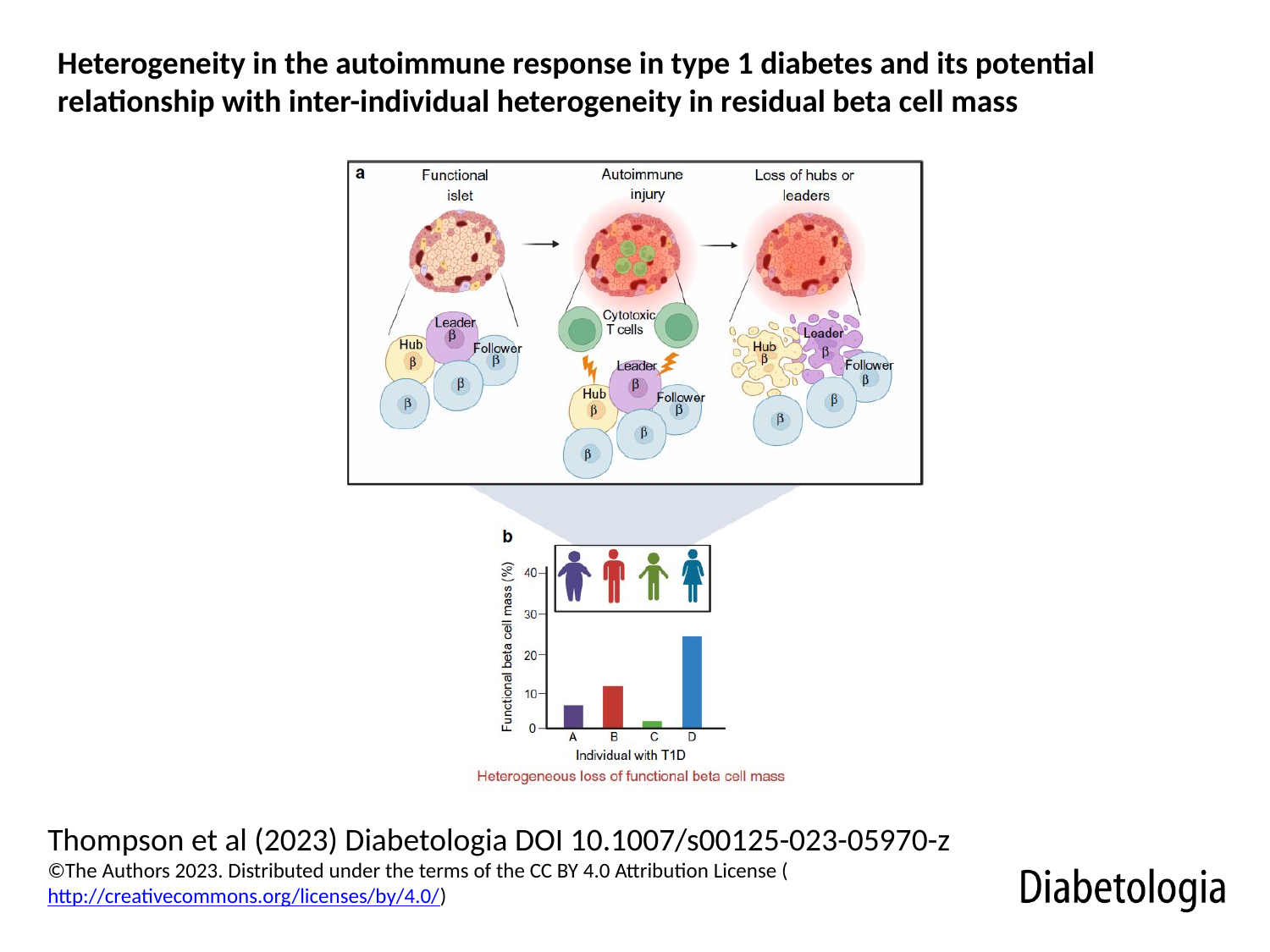

Heterogeneity in the autoimmune response in type 1 diabetes and its potential relationship with inter-individual heterogeneity in residual beta cell mass
Thompson et al (2023) Diabetologia DOI 10.1007/s00125-023-05970-z
©The Authors 2023. Distributed under the terms of the CC BY 4.0 Attribution License (http://creativecommons.org/licenses/by/4.0/)
